# Enhanced spin-phonon-electronic coupling in a 5*d* oxide

**DOI:** 10.1038/ncomms9916

**Published:** 2015-11-26

**Authors:** S. Calder, J. H. Lee, M. B. Stone, M. D. Lumsden, J. C. Lang, M. Feygenson, Z. Zhao, J.-Q. Yan, Y. G. Shi, Y. S. Sun, Y. Tsujimoto, K. Yamaura, A. D. Christianson

**Affiliations:** 1 Quantum Condensed Matter Division, Oak Ridge National Laboratory, Oak Ridge, Tennessee 37831, USA; 2 Materials Science and Technology Division, Oak Ridge National Laboratory, Oak Ridge, Tennessee 37831, USA; 3 School of Energy and Chemical Engineering, Ulsan National Institute of Science and Technology (UNIST), UNIST-gil 50, Ulsan 689-798, Republic of Korea; 4 Advanced Photon Source, Argonne National Laboratory, Argonne, Illinois 60439, USA; 5 Chemical and Engineering Materials Division, Oak Ridge National Laboratory, Oak Ridge, Tennessee 37831, USA; 6 Department of Materials Science and Engineering, University of Tennessee, Knoxville, Tennessee 37996, USA; 7 Institute of Physics, Chinese Academy of Sciences, Beijing 100190, China; 8 Superconducting Properties Unit, National Institute for Materials Science, 1-1 Namiki, Tsukuba, Ibaraki 305-0044, Japan; 9 Materials Processing Unit, National Institute for Materials Science, 1-2-1 Sengen, Tsukuba, Ibaraki 305-0047, Japan; 10 Graduate School of Chemical Sciences and Engineering, Hokkaido University, North 10 West 8, Kita-ku, Sapporo, Hokkaido 060-0810, Japan; 11 Department of Physics and Astronomy, University of Tennessee, Knoxville, Tennessee 37996-1200, USA

## Abstract

Enhanced coupling of material properties offers new fundamental insights and routes to multifunctional devices. In this context 5*d* oxides provide new paradigms of cooperative interactions that drive novel emergent behaviour. This is exemplified in osmates that host metal–insulator transitions where magnetic order appears intimately entwined. Here we consider such a material, the 5*d* perovskite NaOsO_3_, and observe a coupling between spin and phonon manifested in a frequency shift of 40 cm^−1^, the largest measured in any material. The anomalous modes are shown to involve solely Os–O interactions and magnetism is revealed as the driving microscopic mechanism for the phonon renormalization. The magnitude of the coupling in NaOsO_3_ is primarily due to a property common to all 5*d* materials: the large spatial extent of the ion. This allows magnetism to couple to phonons on an unprecedented scale and in general offers multiple new routes to enhanced coupled phenomena in 5*d* materials.

## Abstract (web summary)

Transition metal oxides with 5*d* ions present novel emergent behaviour based on the enhanced coupling of material properties compared to those with 3*d* ions. Here, the authors demonstrate a large spin-phonon coupling in NaOsO_3_ which results from a large Os–O electronic orbital overlap.

## Graphical abstract

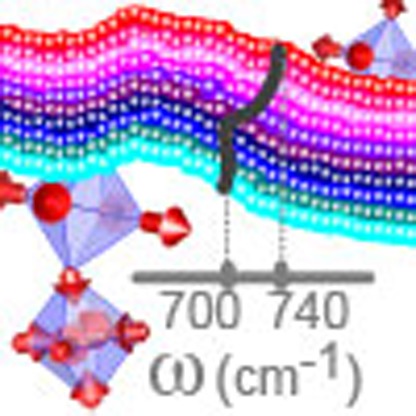

Transition metal oxides with 3*d* ions host a remarkable variety of intriguing phenomena, such as unconventional superconductivity, multiferroic behaviour, colossal magnetoresistance and the Mott metal–insulator transition (MIT)^1^. These properties arise from the strong electron correlations and localized orbitals characteristic of 3*d* ions. Materials with 5*d* ions reside in an alternative regime of intermediate electron correlations, extended orbitals, enhanced spin-orbit coupling (SOC) and large crystalline electric field splitting. The competition and cooperation of these new sets of interactions can drive the emergence of novel behaviour beyond that possible in 3*d*-based materials^2^,^3^. This is manifested in the insulating states of iridates and osmates. In Sr_2_IrO_4_, (ref. 4) and other iridates^5^,^6^, a Mott-like insulating state exists due to enhanced SOC creating a half-filled *J*_eff_=1/2 electronic band that can be split by even the reduced on-site Coulomb interactions of 5*d* ions. Conversely the neighbouring osmate NaOsO_3_, that we focus on here, is believed to host a Slater MIT with behaviour that falls outside the Mott–Hubbard paradigm successfully employed to describe 3*d* transition metal oxides^7^,^8^,^9^,^10^. In the case of a Slater MIT it is the onset of magnetic order and the accompanying creation of a periodic potential that acts as a direct and continuous tuning parameter between metallic and insulating states. The interactions within the 5*d*^3^ ion in NaOsO_3_ appear central to the occurrence of the MIT, with the first proposed three-dimensional Slater candidate Cd_2_Os_2_O_7_ sharing the same 5*d*^3^ electronic ground state.

Here we access the collective excitations and fundamental interactions through the Slater MIT in NaOsO_3_ by probing the phonon modes. Our experimental and theoretical results reveal a high degree of cooperation between the magnetic structure, lattice and electronic conductivity that results in a spin-phonon-electronic coupled transition. The magnitude of the phonon coupling is anomalously large leading us to consider and contrast our results with 3*d* transition metal oxides, where investigating spin-phonon coupling has proven extremely useful in understanding multiferroics^11^,^12^, systems with the same perovskite structure as NaOsO_3_, as well as in a variety of other systems, including high temperature superconductors^13^. The largest reported phonon shift in a perovskite is found in (Sr,Ba)MnO_3_ with a value of Δ*ω*=25 cm^−1^ in the TO_1_ polar phonon^14^,^15^. While the investigations of phonon modes in the context of 5*d* materials is currently limited, it was recently found that the mixed 3*d*–5*d* half-metal double perovskite Ba_2_FeReO_6_ hosts a dramatic spin-electron-phonon coupling as evidenced by a phonon shift of Δ*ω*=30 cm^-1^, (ref. 16) the largest ever reported prior to our present work on NaOsO_3_. The phonon shift in Ba_2_FeReO_6_ is reported as being directly linked to the interaction between the 3*d* and 5*d* ions. Conversely, we show here that 5*d* ions alone can produce even larger spin-phonon shifts. By considering the various competing mechanisms in NaOsO_3_, including the electronic changes at the MIT and structurally driven charge disproportionation, we find the microscopic behaviour to be driven by the G-type magnetic structure that orders in the perovskite structure. The enhanced nature is promoted by the extended orbitals of the 5*d* ion that supports strong coupling between the magnetic superexchange and phonon vibrations. By contrasting our results with measurements on Cd_2_Os_2_O_7_, that show a much reduced spin-phonon shift, we consider the key ingredients required to achieve even larger spin-phonon coupling in general in further systems.

## Results

### Measurement of anomalous spin-phonon coupling in NaOsO_3_

**Figure 1:**
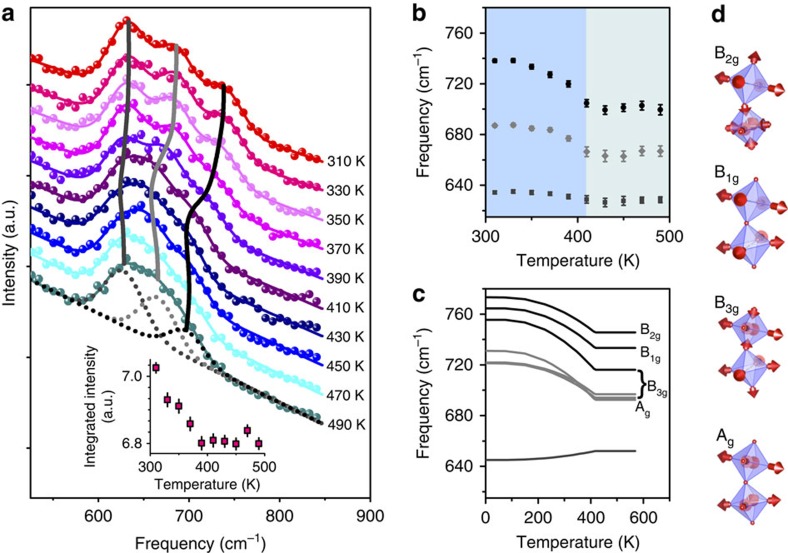
Measured and calculated phonon modes in NaOsO_3_ through the magnetic MIT. (**a**) Thermal evolution of the phonon mode density of states measured with inelastic neutron scattering through the magnetic Slater MIT temperature of 410 K. Three modes are resolvable between 550 and 800 cm^−1^ at all temperatures. The data (filled circles) were modelled (coloured lines) to three Gaussian lineshapes with the width of the energy resolution (∼15 cm^−1^ full width at half maximum). The three resolvable modes are shown for the 490 K data by the grey dashed lines. The different temperature measurements are shown offset in intensity to aid comparison. The three grey vertical lines indicate the frequencies from the Gaussian fits to the spectra for each temperature. Inset reveals an abnormal intensity increase with decreasing temperature of the integrated intensity over the region 550–800 cm^−1^. (**b**) The shaded regions distinguish the low temperature magnetic-insulating and high temperature non-magnetic metallic phases in NaOsO_3_. The measured phonon mode frequencies obtained from inelastic neutron scattering and (**c**) from DFT calculations both show strong agreement and reveal a phonon shift at the Slater transition of Δ*ω*=40 cm^−1^. The DFT calculations allow assignment of the responsible modes, as indicated. The breathing modes occur at higher frequencies than the asymmetric stretching, with B_2g_ occurring at the highest frequency. (**d**) The separate distortions, that all involve Os–O interactions, are shown with the directions indicated by the red arrows. A_g_ (in phase) and B_3g_ (out of phase) correspond to asymmetric stretching. B_1g_ (in phase) and B_2g_ (out of phase) represent symmetric stretching breathing modes. Error bars throughout the figure represent the s.d. in the data fitting procedure.

To follow the behaviour of collective excitations in NaOsO_3_ through the magnetic MIT we performed inelastic neutron scattering (INS) measurements. Figure 1a shows the key result of the temperature dependence of the phonon density of states (pDOS) whose peaks are related to the underlying phonon modes. We focus on the region around 700 cm^−1^ that covers the essential physics of interest. The full spectrum is shown in Supplementary Fig. 1. Three distinct resolution limited peaks in the pDOS are observed around 700 cm^−1^ and fitting these each to a Gaussian, as shown in Fig. 1a, allows the energy of the modes to be followed with temperature. The key result of a pronounced phonon frequency shift is observed in Fig. 1a,b. Moreover there is an anomalous and counterintuitive intensity increase with decreasing temperature through the MIT as shown inset Fig. 1a considering the entire range of 550–800 cm^−1^. The results are significant in several regards. First, the onset of the phonon mode shift is concurrent with the magnetic MIT in NaOsO_3_ at 410 K, indicating a coupling of the phonons to the magnetic and electronic transitions. Second, the phonons show a shift of Δ*ω*=40 cm^−1^, the largest measured in any material for a spin-phonon coupled transition.

To begin to understand the microscopic origin of the behaviour in NaOsO_3_ we consider the role of the MIT with complimentary neutron measurements on Cd_2_Os_2_O_7_. Cd_2_Os_2_O_7_ was chosen since it has the same 5*d*^3^ electronic configuration of the Os^5+^ ion and hosts a magnetic MIT that is very similar to NaOsO_3_, with current debate as to whether the mechanism is Slater or Lifshitz^17^,^18^. The inelastic neutron measurements through the magnetic MIT in Cd_2_Os_2_O_7_ are shown in Supplementary Fig. 2. While there is an apparent phonon shift at the magnetic transition the value of Δ*ω*=4 cm^−1^ is much reduced from NaOsO_3_. The disparate results for NaOsO_3_ and Cd_2_Os_2_O_7_ indicate that the underlying mechanism for the anomalously large behaviour in NaOsO_3_ cannot be attributed to the occurrence of the MIT, since both host similar MITs with similar energy scales. Instead, as we support with further results and calculations, the microscopic mechanism is related to the magnetic ordering and lattice topology of NaOsO_3_.

Considering a further pertinent material, the 3*d–*5*d* material Ba_2_FeReO_6_ that showed a phonon shift of Δ*ω*=30 cm^−1^, we note this occurred concurrent with a structural symmetry change^16^. No symmetry change has been detected in NaOsO_3_ (refs 8, 9). However, to explore this possibility further we performed detailed neutron pair density functional measurements through the Slater MIT, see Supplementary Figure 3, and found no local symmetry change. Hence, the enhanced spin-phonon coupling does not appear to arise due to static long or short range lattice distortions in NaOsO_3_.

### Theoretical demonstration of spin-phonon shift

To gain a microscopic insight into the origin of the anomalous phonon mode behaviour in NaOsO_3_ and disentangle the myriad of competing interactions at the magnetic and electronic transition we performed detailed density functional theory (DFT) calculations. The DFT results show the same three phonon modes observed with INS between 600 to 900 cm^−1^, see Fig. 1c, and as expected for the orthorhombic structure in NaOsO_3_ these are themselves composed of three branches, unresolvable in the current powder INS measurement. The theoretical shift is in very close agreement with the measured value of Δ*ω*=40 cm^−1^. This indicates the calculations that probe only the Brillouin zone center accurately reproduce the essential physics of the system as measured by neutrons that probe the entire Brillouin zone. The thermal behaviour was captured in the calculations by increasing the magnetic moment <**S**_i_.**S**_j_> to reproduce the onset of G-type antiferromagnetic order in NaOsO_3_ with the predicted magnetic ordering at 415 K very close to the 411 K observed experimentally. The DFT results, with the need to include magnetism, immediately indicate that the mechanism of the phonon shift is entwined with the onset of magnetic order.

### Octahedral B_2g_ breathing mode and charge disproportionation

**Figure 2:**
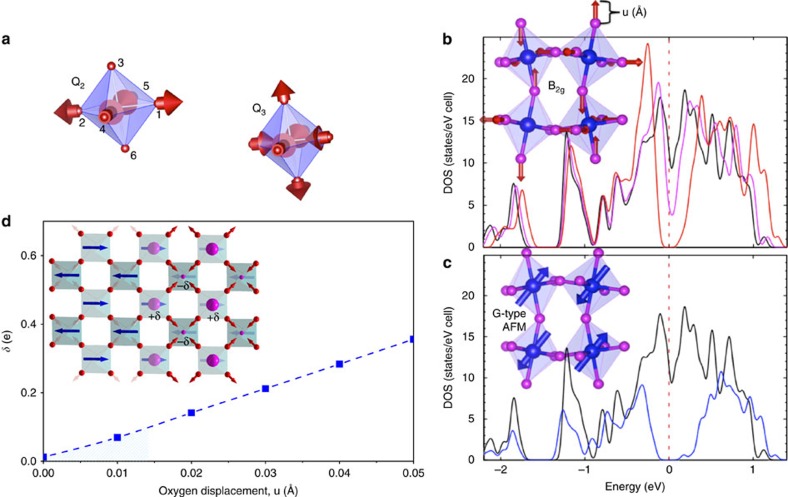
Charge disproportionation created by B_2g_ breathing mode. (**a**) The static octahedral distortion can be quantified with parameters *Q*_2_ and *Q*_3_ that represent the degree of octahedral anisotropy defined as *Q*_2_=(*x*_1_−*x*_4_−*y*_2_+*y*_5_)/√2 and *Q*_3_=(2*z*_3_−2z_6_−*x*_1_+*x*_4_−*y*_2_+*y*_5_)/√6, where *x*, *y* and *z* are the oxygen positions labelled 1−6. The red arrows indicate the directions of Os–O distortions. Unusually *Q*_2_ and *Q*_3_ both show reduced values below the Slater MIT indicating the octahedra become more isotropic at lower temperature. (**b**) This counterintuitive behaviour is compatible with the symmetric three-dimensional B_2g_ distortion, depicted by red arrows, increasing in the low temperature insulating regime. The solid lines are DOS calculations for oxygen displacements (u), within the paramagnetic regime, of *u*=0 Å (black line), *u*=0.1 Å (magenta line) and *u*=0.2 Å (red line). For the large oxygen displacement of *u*=0.2 Å, much beyond that accessed in our measurements, the breathing distortion can open a band gap as revealed in the DOS from DFT calculations. (**c**) We stress by reproducing published results^8^ that it is solely the onset of G-type magnetic ordering that opens the gap via the Slater mechanism in NaOsO_3_. No oxygen displacement, *u*=0 Å, (black line) shows no gap in the DOS whereas an insulating gap is created for G-type antiferromagnet (AFM) order (blue line). This ordering is indicated by the blue arrows. (**d**) Although the required u displacement is too large to drive the MIT in NaOsO_3_, it creates charge disproportionation (δ(e)) on the Os ion. As shown schematically the G-type antiferromagnetic ordering (blue arrows) and periodic expansion/contraction of the B_2g_ breathing mode ordering are isosymmetric in NaOsO_3_. A consequence of the static ordering of the octahedra, as shown in frozen DFT calculations, is the creation of charge disproportionation, indicated by the creation of +*δ* and –*δ* ordering (magenta sphere). The predicted value in NaOsO_3_, *δ*(e), is indicated by the blue shaded region.

The calculations reveal all of the phonon modes and are shown in Fig. 1d. They all correspond to Os–O vibrations, specifically breathing modes B_1g_ (in phase) and B_2g_ (out of phase) and two asymmetric Jahn–Teller stretching modes A_g_ (in phase) and B_3g_ (out of phase). To reveal the role of these modes in NaOsO_3_ we begin by first considering the static behaviour of the octahedra and propensity towards Jahn–Teller distortion. This can be quantified by introducing parameters *Q*_2_ and *Q*_3_, which are shown schematically in Fig. 2a, and defined as *Q*_2_=(*x*_1_−*x*_4_−*y*_2_+*y*_5_)/√2 and *Q*_3_=(2*z*_3_−2*z*_6_−*x*_1_+*x*_4_−*y*_2_+*y*_5_)/√6, where *x*, *y* and *z* are the oxygen positions^19^. Thereby the values of *Q*_2_ and *Q*_3_ reveals the degree of static octahedral anisotropy, with the larger the value the more distorted the octahedra. Calculations from experimentally determined atomic parameters for NaOsO_3_ (ref. 9) reveal *Q*_2_ and *Q*_3_ to be small at all temperatures, but counter intuitively decrease through the Slater MIT. Specifically, at 500 K *Q*_2_=0.0114(15) a.u. and at 300 K *Q*_2_=0.0035(11) a.u. While at 500 K *Q*_3_=0.0171(18) a.u and at 300 K *Q*_3_=0.0114(14) a.u. Therefore this reveals that in NaOsO_3_ the octahedra actually become more isotropic in three dimensions within the low temperature insulating regime. This behaviour is at odds to the normal Jahn–Teller distortions of increased anisotropy and does not favour the asymmetric stretching modes A_g_ and B_3g_. Instead the increased static octahedral isotropy is more conducive to the symmetric breathing distortions B_1g_ and B_2g_. Indeed the abnormal behaviour of the intensity increase of the pDOS, in inset of Fig. 1a, is consistent with an increase in vibration with decreasing temperature, counter to usual thermal behaviour. This appears most pronounced at the highest frequency, which corresponds to the breathing mode B_2g_ and consequently appears central to the behaviour of NaOsO_3_.

Considering the B_2g_ mode further we find that sufficiently large breathing distortions of the octahedra, much larger than accessed in our measurements, offers a potential route to opening the insulating gap in the paramagnetic regime, see Fig. 2b. Our frozen DFT results show that in the perovskite structure of NaOsO_3_ the gap opening can occur since the octahedral breathing causes neighbouring octahedra to expand/contract that in turn creates a periodic charge disproportionation on the Os ion. In addition there is apparent isosymmetric ordering and coupling between the G-type antiferromagnet and octahedral B_2g_ mode ordering (behaviour shown schematically in Fig. 2d). We note that no other phonon distortion produces similar periodic ordering or routes to open a gap. For the B_2g_ mode to create a gap the minimum required oxygen displacement is *u*=0.2 Å, see Fig. 2b. This corresponds to 10% of the actual Os–O bond distance and therefore it is too large to allow this mechanism to drive the MIT in NaOsO_3_. However, statically, while not opening a gap the periodic octahedral breathing ordering creates a strong charge disproportionation of Δ*δ*/Δ*u*=7.0 e/Å in the lattice due to the change of the electronic potential around the Os ion and places the system on the verge of a MIT. For example considering a nominal Os–O phonon vibration displacements of the order 0.01 Å as occurring in NaOsO_3_ then the dynamic charge disproportionation will be ∼0.01e. This indicates that suitable control of the octahedra via pressure or strain is a potential route to tune the MIT in NaOsO_3_. We stress that, as shown in Fig. 2c that substantiates earlier work^8^, it is the onset of antiferromagnetic order alone that creates the insulating gap via the Slater mechanism.

### Suppressed role of SOC in NaOsO_3_

While SOC is often attributed to anomalous behaviour of 5*d* materials, in NaOsO_3_ the 5*d*^3^ t_2g_-degenerate ground state will suppress the effective SOC^20^. We nevertheless addressed the role of SOC with X-ray absorption near edge spectroscopy that allows a quantitative comparison with SOC enhanced iridates. As expected our results indicate SOC does not play a dominant role in the behaviour of NaOsO_3_ as discussed in the Supplementary Material (Supplementary Fig. 4 and Supplementary Note 1). Our first-principle results additionally show the large coupling without SOC.

### Coupling of lattice, magnetic order and MIT

**Figure 3:**
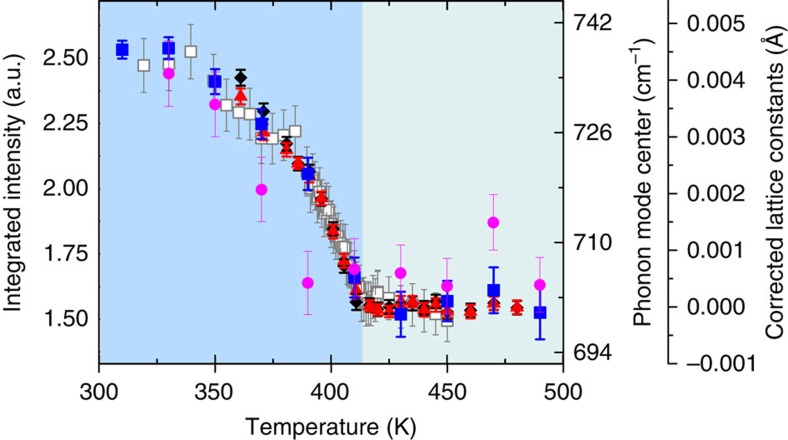
Phonon, lattice and magnetic degrees of freedom couple through the Slater MIT in NaOsO_3_. The measured temperature dependence of the phonon mode frequency shift (blue square), the [110] magnetic Bragg reflection intensity^9^ (white square), and the *a* (black diamond) and *c* (red triangle) lattice constants in NaOsO_3_ (altered from ref. 9) show a direct scaling with temperature through the Slater MIT due to spin-phonon-electronic coupling. The lattice parameters have been corrected by removing a constant sloping thermal background. Error bars throughout the figure represent the s.d. in the data fitting procedure. The shaded regions distinguish the low temperature magnetic-insulating and high temperature non-magnetic metallic phases in NaOsO_3_.

The coupled properties in NaOsO_3_ are illustrated in Fig. 3 where experimentally there is a direct scaling of the structural anomaly of the lattice constants, phonon mode shift, magnetic moment, with the MIT qualitatively following a similar trend. This reveals a high degree of cooperation in NaOsO_3_ via spin-phonon-electronic coupling. While the realization of numerous overlapping phenomena is currently rare it is likely that additional 5*d* materials will host similar rich phase diagrams with the prospect of enhanced magnitudes.

## Discussion

We have presented both experimental and theoretical results that show an enhanced phonon shift in NaOsO_3_, which along with the concurrent magnetic MIT creates a spin-phonon-electronic transition above room temperature. Considering the collective results we argue that the occurrence of the anomalous spin-phonon behaviour is a direct consequence of the extended 5*d* orbitals coupling to the magnetic structure via the Os–O–Os superexchange interactions on an unprecedented scale and is not driven by the MIT or structural anomalies. The central role of magnetism is emphasized in our DFT calculations that require the inclusion of magnetic order to reproduce the experimental phonon shift. However, as the reduced spin-phonon shift in Cd_2_Os_2_O_7_ attests, the presence of 5*d* magnetic order alone is not sufficient to induce enhanced coupled phenomena. Instead, within 5*d* systems, it is not just the onset of magnetic ordering but the specific type of magnetic structure and the lattice it resides on that supports enhanced spin-phonon coupling.

By considering the specific modes in NaOsO_3_ the B_2g_ breathing mode emerges as central to the behaviour. The mode is characterized by expansion/contraction of neighbouring octahedra that in NaOsO_3_ can statically order in a periodic way, isosymmetric with the magnetic spins (G-type antiferromagnet), with every nearest neighbouring octahedra oppositely expanded/contracted (see Fig. 2). The breathing mode ordering causes charge disproportionation that grows as the B_2g_ vibrations increase within the low temperature insulating phase and can promote the opening of a charge gap for sufficiently large vibrations. However, the distortion under the experimental conditions we measured falls well short of the value required to drive a MIT via this alternative non-magnetic mechanism in NaOsO_3_. Instead, once the insulating gap develops via solely the magnetic Slater mechanism, the breathing mode becomes favourable and the structure subtly alters allowing the octahedra to become more isotropic as a route to increase the symmetric breathing mode and consequently enhance the insulating state.

All of the modes uncovered that show an anomalous shift (B_1g_, B_2g_, A_g_ and B_3g_) are characterized by simultaneous bond-stretching/shrinking between the oxygen and osmium ions of the OsO_6_ octahedra. Therefore as these modes vibrate they change the O–Os–O wavefunction overlap. Since the magnetism in NaOsO_3_ is mediate by the superexchange interaction this provides a direct route to couple to the Os–O modes. In the non-magnetic regime the frequency of the Os–O modes do not change, as expected. However, as the magnetic order develops via the Os–O superexchange interactions this couples to the Os–O modes. The degree of the coupling is governed by the 5*d*(Os)-2*p*(O) orbital overlap, which is much larger in 5*d* oxides compared to analogous 3*d* systems. The consequence is that in NaOsO_3_ the large wavefunction overlap results in a magnification of the spin-phonon coupling, with the expectation that similar behaviour will be found in further 5*d*-based systems since the extended orbitals are an intrinsic property. With this expectation for designing 5*d* materials with strong coupling in mind the reduced spin-phonon coupling in Cd_2_Os_2_O_7_ provides useful insights. The reduced spin-phonon magnitude in Cd_2_Os_2_O_7_ naturally occurs by considering the differences in the lattice topology, specifically the Os–O–Os bond that mediates the emergent behaviour. While the Os–O–Os bond distance is nearly identical in NaOsO_3_ and Cd_2_Os_2_O_7_ the bond angles diverge. In NaOsO_3_ it is 155°, whereas in Cd_2_Os_2_O_7_ it is 137°, appreciably further from 180°. Consequently the propagation of …–Os–O–Os–… vibrations throughout the lattice can be sufficiently suppressed within the pyrochlore structure resulting in a reduced coupling of phonons to the magnetic interactions and a smaller, although still finite, spin-phonon coupling. Collectively our results indicate that new cases of similarly enhanced spin-phonon coupling, along with further coupled phenomena, should be found in cubic 5*d* perovskites with near ideal 180° Os–O–Os bonds.

## Methods

### Synthesis

Polycrystalline samples of NaOsO_3_ were prepared using a high pressure solid state synthesis with pressures of 6 GPa, as described in ref. 8. Polycrystalline Cd_2_Os_2_O_7_ was prepared with isotopic ^114^Cd for neutron measurements to negate the extremely high neutron absorption of standard Cd using sold state techniques from ^114^CdO and OsO_2_ powders.

### Inelastic neutron scattering

Inelastic neutron scattering measurements were performed on the ARCS and SEQUOIA spectrometers at the spallation neutron source on a 5-g polycrystalline sample of NaOsO_3_ and 7-g polycrystalline sample of Cd_2_Os_2_O_7_, respectively. The NaOsO_3_ sample was loaded into a vanadium can and measurements performed between 300 and 500 K using an incident energy of 120 meV. The Cd_2_Os_2_O_7_ sample was measured in an Al can from 150 to 250 K using an incident energy of 100 meV. Corrections for the Bose factor, where appropriate, were performed using the DAVE software^21^.

### DFT

First-principles calculations were performed using density functional theory within the generalized gradient approximation GGA+U method with the Perdew–Becke–Erzenhof parameterization as implemented in the Vienna *ab initio* Simulation Package (VASP 5.3)^22^. Theoretical details for spin-phonon coupling are described in ref. 23. We use the Dudarev^24^ implementation with on-site Coulomb interaction U=1.7 eV and on-site exchange interaction *J*_H_=1 eV, so *U*_eff_=0.7 eV to treat the localized d electron states in Os. Within GGA+U, this small U gives excellent agreement between the experimental Neel temperature (*T*_N_=411 K) and calculated one (*T*_N,MFT_=415 K) in mean-field approximation. The projector augmented wave potentials^25^ explicitly include 9 valence electrons for Na (2*s*^2^ 2*p*^6^ 3*s*^1^ ), 14 for Os (5*p*^6^ 5*d*^5^ 6*s*^2^) and 6 for oxygen (2*s*^2^ 2*p*^4^). To capture spin-phonon coupling with respect to temperature we employed the method successfully used for various magnetic perovskites^19^,^23^.

Our calculations use the harmonic approximation throughout. This route is supported by the experimental observation that the peak widths are resolution limit and do not show any broadening that would be associated with anhormonicity.

### Neutron pair density function

Neutron pair density functional measurements were performed on the Nanoscale-Ordered Materials Diffractometer (NOMAD) beamline at the spallation neutron source on a powder sample of NaOsO_3_ from 370 to 460 K. The data were analyzed and modelled with pdfgui^26^.

### X-ray absorption near edge spectroscopy

X-ray absorption measurements were performed at the advanced photon source on sector 4-ID-D. Spectra were collected at room temperature on a powder sample (∼100 mg) in transmission mode through the Os L_2_ and L_3_ edges. Analysis was performed with the Athena software^27^.

## Additional information

**How to cite this article:** Calder, S. *et al*. Enhanced spin-phonon-electronic coupling in a 5*d* oxide. *Nat. Commun.* 6:8916 doi: 10.1038/ncomms9916 (2015).

## Supplementary Material

Supplementary Information Supplementary Figures 1-4, Supplementary Note 1 and Supplementary References
