# Supplementary material for: Enhanced spin-phonon-electronic coupling in a 5d oxide
Source: Nat Commun. 2015 Nov 26;6:8916. doi: 10.1038/ncomms9916 (PMC4674761; doi:10.1038/ncomms9916)
Supplement: Supplementary Information — Supplementary Figures 1-4, Supplementary Note 1 and Supplementary References [file ncomms9916-s1.pdf]

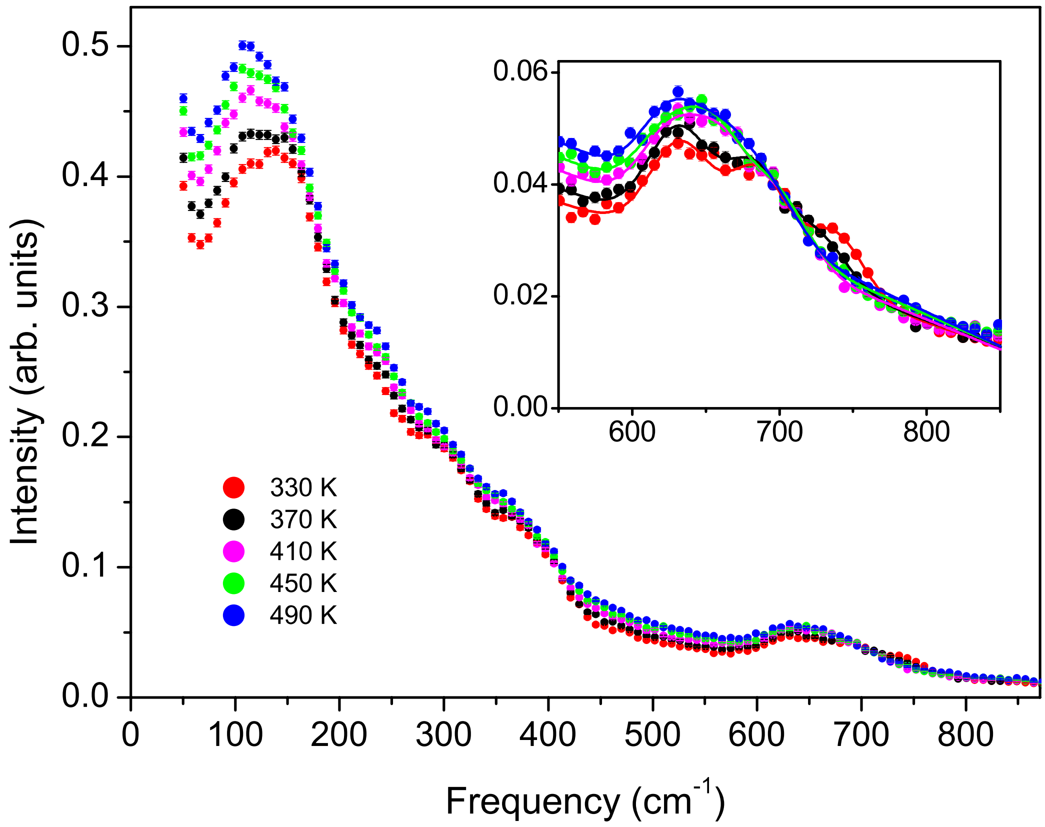


**Supplementary Figure 1 | Inelastic neutron scattering spectra through the magnetic MIT temperature in $NaOsO_3$.** Inelastic neutron scattering measurements with an incident energy of 120 meV over the full range of energy through the temperature region of interest. The data is plotted without correcting for the Bose factor. The only energy at which the intensity increases at low temperature is around the frequency we have focused on, ~750 cm$^{-1}$. The large intensity variation at low frequency, particularly around 100 cm$^{-1}$, is expected and is a consequence of increased thermal population with increasing temperature.

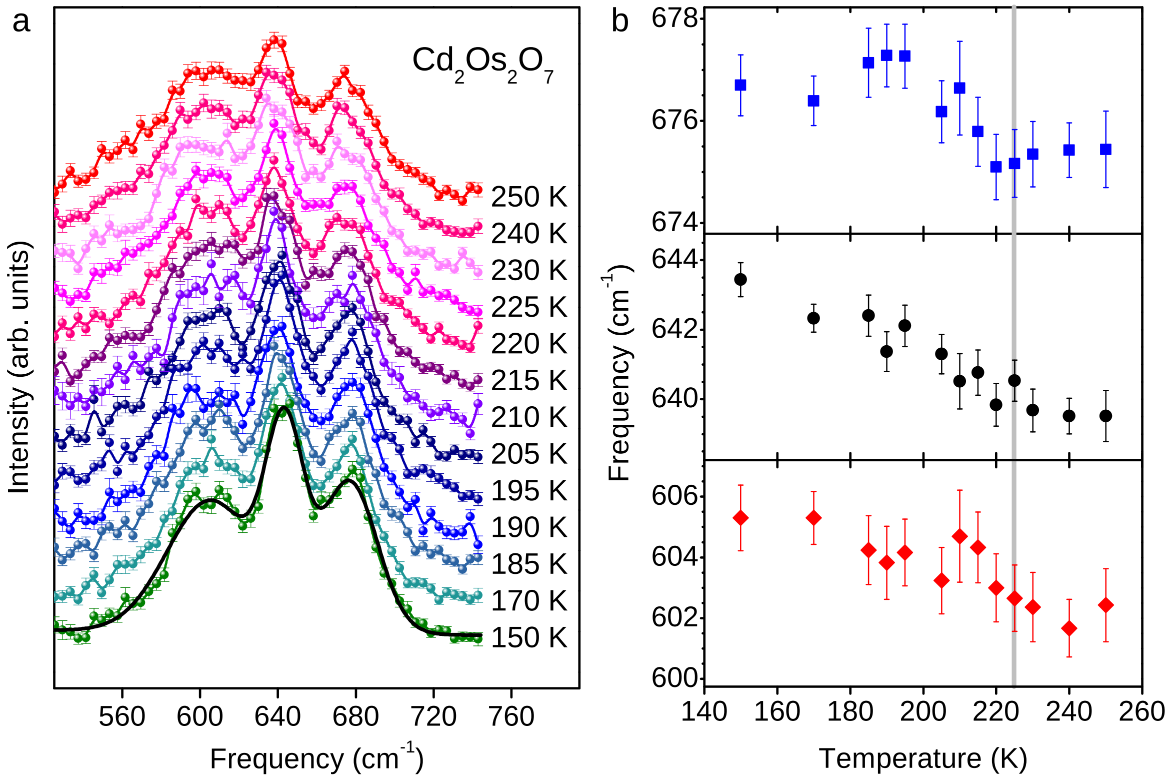


**Supplementary Figure 2 | Inelastic neutron scattering measurements of the pDOS in $Cd_2Os_2O_7$. (a)** Neutron measurements were performed through the magnetic MIT temperature of 225 K in $Cd_2Os_2O_7$. For these measurements $^{114}Cd$ was used to overcome the significant neutron absorption of elemental Cd. The observed phonon scattering in the similar region of interest to $NaOsO_3$ was fit to three Gaussians of FWHM of the experimental resolution. The different temperature measurements have been offset in the y-axis to aid comparison. The solid black line shows the fit to the 150 K based on three Gaussians. **(b)** The frequency variation of the three modes shows an anomaly at the magnetic MIT (225 K), the solid grey line indicates this temperature. For T>225 K, within error, the positions of the peaks remain constant. However, below the magnetic MIT the peaks shift appreciably, with a maximum shift of $\Delta\omega=4$ $cm^{-1}$. This shifting, indicative of spin-phonon coupling, is significantly reduced compared to $NaOsO_3$. Error bars throughout the figure represent the s.d. in the data fitting procedure.

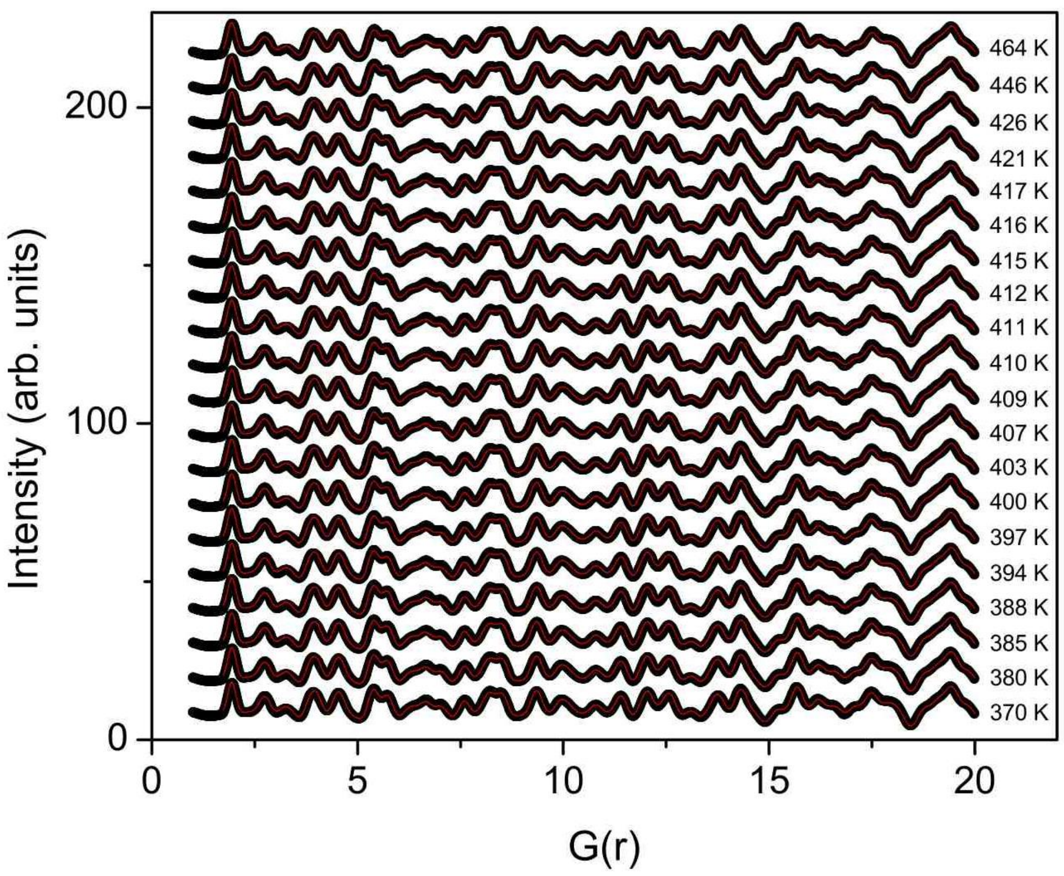


**Supplementary Figure 3 | Neutron PDF measurements through the magnetic MIT in $NaOsO_3$.** Neutron PDF data from 370 K to 464 K are offset in intensity and fit to the Pnma structure using the PDFgui software.[1] The results reveal no local symmetry change occurs through the 410 K Slater transition in $NaOsO_3$. Therefore on both a local and global level $NaOsO_3$ undergoes no structural symmetry in the temperature range of interest. While a symmetry change does not occur, the lattice reflects the magnetic MIT with a small shift in the a and c lattice constants of under 0.1%, as previously reported.[2] The origin of this is due to a subtle alteration of the octahedra as they become more isotropic due to the breathing phonon mode, as discussed in the main text.

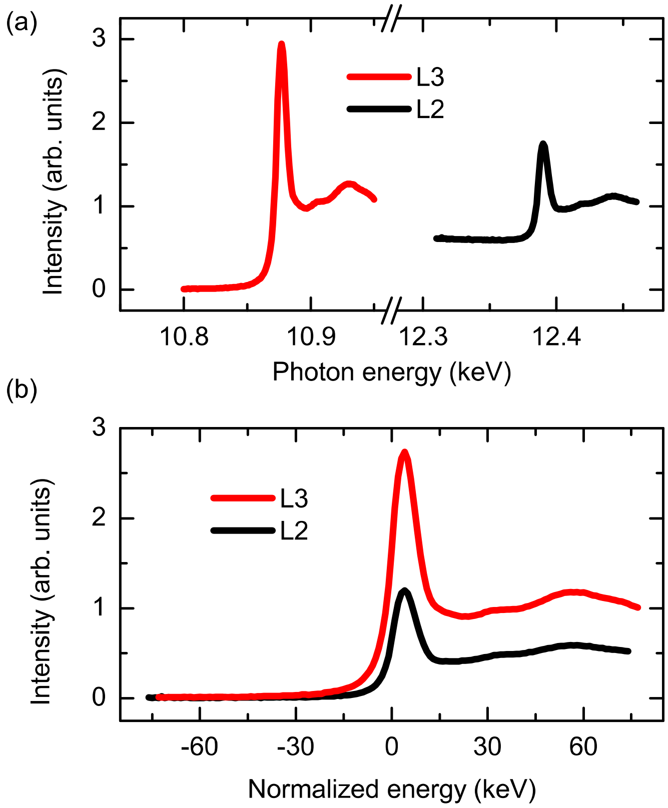


**Supplementary Figure 4 | X-ray near edge absorption spectroscopy (XANES) measurements investigating SOC.** **(a)** XANES results at the $L_2$ and $L_3$ absorption edges for $NaOsO_3$. **(b)** The XANES results are shifted in energy to be centered at zero on their respective absorption energies and the intensities normalized to allow a comparison. The ratio of the integrated intensities is 2.6:1.

## Supplementary Note 1

**Role of SOC in $NaOsO_3$.** The significant enhancement of SOC in going from 3d to 5d ions offers a potential explanation for anomalous behavior between 3d and 5d analogues. Previous experimental and theoretical investigations, however, have found that SOC does not play a dominant role in the electronic ground state of $NaOsO_3$, [2,3] distinct from the neighboring iridates. Nevertheless SOC is often employed and cited as a significant factor in general in 5d oxides and therefore we directly addressed the role on a quantitative level to rule out SOC as being a principle factor at play in the anomalous phonon behavior in $NaOsO_3$. An important experimental tool in this regard to investigate the role of SOC, and allow comparisons between different materials and ions, is x-ray absorption near edge spectroscopy (XANES). Results of such measurements on $NaOsO_3$ are shown in Supplementary Figure 4. A statistical $L_3$:$L_2$ white-line branching ratio BR of 2:1 is expected in XANES, independent of the electron occupancy of the ion under investigation. The measured ratio of integrated intensities for the $L_3$:$L_2$ edge is 2.6:1. This allows a direct comparison of $Os^{5+}$ in $NaOsO_3$ with $Ir^{4+}$ in $Sr_2IrO_4$ and $BaIrO_3$ that show significant SOC effects and additionally with other 5d ions, such as Re, that show reduced SOC effects. The BR of 2.6:1 in $NaOsO_3$ contrasts with 4:1 for $BaIrO_3$, with the large deviation from 2:1 in iridates presented as direct proof of strong spin-orbit coupling.[4] To directly compare our results we extract the ground state expectation value of the angular part of the spin-orbit coupling $\langle \mathbf{L.S} \rangle$, through BR = (2+r)/(1-r), where $r = \langle \mathbf{L.S} \rangle / \langle n_h \rangle$ and $n_h$ is the number of holes in the 5d manifold. Using the XAS branching ratio of 2.6 and $n_h=7$ for $Os^{5+}$ gives $\langle \mathbf{L.S} \rangle \approx 1$ in units of $\hbar^2$. This contrasts with $\langle \mathbf{L.S} \rangle \approx 2$ for $Ir^{4+}$ in $BaIrO_3$ from XANES using the same method with BR=4 and $n_h=5$.[4] The reduced value of the expectation value of the SOC in $NaOsO_3$, compared to $BaIrO_3$, indicates SOC is not the explanation for the observed spin-phonon behavior.

**Supplementary References**

1. Ravel, B. and Newville, M., *ATHENA, ARTEMIS, HEPHAESTUS: data analysis for X-ray absorption spectroscopy using IFEFFIT*, Journal of Synchrotron Radiation **12**, 537–541 (2005).
2. Calder, S. et al., Magnetically driven metal-insulator transtion in $NaOsO_3$. Phys. Rev. Lett. **108**, 257209 (2012).
3. Shi, Y. G. *et al.*, Continuous metal-insulator transition of the antiferromagnetic perovskite $NaOsO_3$. *Phys. Rev. B* **80**, 161104 (2009).
4. Laguna-Marco, M. A. *et al.*, Orbital Magnetism and Spin-Orbit Effects in the Electronic Structure of $BaIrO_3$. *Phys. Rev. Lett.* **105**, 216407 (2010).
